# Use of nomograms based on the one-compartment model in the estimation of area under the blood concentration–time curve of vancomycin: a retrospective cohort study

**DOI:** 10.1007/s00228-025-03885-9

**Published:** 2025-08-04

**Authors:** Go Okamoto, Aya Kawakami, Daisuke Ito, Miki Sawai, Kanae Okano, Emiko Kobayashi

**Affiliations:** Department of Pharmacy, Tokyo Women’s Medical University, Yachiyo Medical Center 477-96 Oowadashinden, Yachiyo Chiba, 276-8524 Japan

**Keywords:** Antibiotics, Infectious diseases, Vancomycin, Therapeutic drug monitoring, Area under the concentration–time curve

## Abstract

**Background:**

Current guidelines for vancomycin dosing and monitoring recommend calculating area under the concentration–time curve (AUC) using a Bayesian approach. However, Bayesian approaches are challenging, requiring specialised software and expertise. Thus, simpler AUC calculation methods are required.

**Aim:**

To develop and validate a nomogram to estimate the AUC of vancomycin based on the mathematical equation of the one-compartment model, and to investigate the clinical applicability of the apparent volume of distribution (Vd_,app_) estimated from the first peak concentration after the first dose as an alternative to the volume of distribution.

**Methods:**

AUC values based on the nomogram and the Bayesian approach were compared in 51 patients who received vancomycin between May 2022 and December 2023. The first peak concentration and steady-state trough concentrations were measured in all patients, and Vd_,app_ was calculated using the following formula: Vd_,app_ = First dose / the first peak concentration. The regression line was estimated using the Passing–Bablok method, and the correlation was evaluated using Kendall’s tau.

**Results:**

The regression line relating the AUC based on the nomogram to the Bayesian approach had a slope of 0.988 (95% CI, 0.855, 1.131) and an intercept of 5.35 (95% CI, − 48.59, 64.26). Kendall’s tau was 0.643 (*P* < 0.01), indicating a positive correlation.

**Conclusion:**

The estimation method utilising AUC nomograms and Vd_,app_ represents a straightforward approach with accuracy comparable to that of the Bayesian method, suggesting its potential for clinical application. However, its implementation in cases of high clearance or in patients with severe infections warrants careful consideration.

**Supplementary Information:**

The online version contains supplementary material available at 10.1007/s00228-025-03885-9.

## Introduction

The 2020 guidelines for vancomycin (VCM) dosing and therapeutic drug monitoring (TDM) recommend area under the concentration–time curve (AUC)-guided TDM instead of steady-state trough concentration (C_trough,ss_)-guided TDM [1]. The target ratio of AUC to minimum inhibitory concentration (AUC/MIC) is 400–600 [1, 2]. The Bayesian approach is recommended for calculating AUC based on population pharmacokinetic (PopPK) parameters and two serum VCM concentrations (C_vcm_) [1, 2]. Although slightly less accurate than two C_vcm_, the Bayesian approach utilising only C_trough,ss_ more effectively reduces the risk of acute kidney injury (AKI) [3, 4]. However, Bayesian approaches are challenging to compute without specialised software or web applications, and professional training is required. Furthermore, widespread implementation of AUC-guided TDM is hindered by the lack of equipment and education [5, 6]. The first-order pharmacokinetic equations to estimate AUC from C_trough,ss_ and C_vcm_ obtained 1–2 h post-infusion at steady-state are recommended as in the Bayesian approach [1, 7], and are equally accurate in estimating AUC [7, 8]. First-order pharmacokinetic equations are readily implementable, but this approach requires two C_vcm_ measurements for each TDM. To mitigate the burden and costs of frequent C_vcm_ measurements, researchers have been investigating methods to estimate the AUC using minimal C_vcm_ data points. One approach based on a one-compartment model equation, an estimation model for volume of distribution (Vd) and C_trough,ss_ (C_trough,ss_-equation approach) has demonstrated accuracy in calculating the AUC [9, 10]. Another approach utilizing AUC estimation nomograms with a single dosage per body weight (BW) and C_trough,ss_, significantly reduced AKI compared to the C_trough,ss_-guided TDM [11]. Importantly, the two pharmacokinetic (PK) parameters required for the C_trough,ss_-equation approach are the elimination rate constant (Ke) and Vd. If Vd is known, then the AUC can be estimated from the C_trough,ss_. While there are several methods of estimating Vd, a practical approximation can be obtained by calculating the apparent Vd (Vd_,app_) as a ratio of the first dose (FD) to the concentration measured 1 h post-infusion (C_peak,first_). Although this introduces some degree of error, it enables a more straightforward AUC calculation.

### Aim

This study aimed to: (1) compare AUC values obtained from a one-compartment model using Vd_,app_ with those from a two-compartment model through simulations; (2) develop a nomograms based on a one-compartment model with intravenous infusion to estimate AUC from Vd and C_trough,ss_; and (3) evaluate the clinical utility of AUC estimates derived using Vd_,app_ and the nomograms by comparison with Bayesian estimate.

### Ethical approval 

The study was approved by the Ethics Committee of Tokyo Women’s Medical University (approval number: 2024–0005). This retrospective study employed an opt-out approach, providing individuals with the opportunity to decline participation, with study information made publicly available on the website of Tokyo Women’s Medical University.

## Method

### Simulation with pseudo-parameters: comparison of AUCs based on one- and two-compartment models

Utilising the previously published PopPK parameters from the Yasuhara model for the Japanese population [12], we generated 5000 sets of pseudo-random data for creatinine clearance (CL_cr_) and for the two-compartment model parameters: clearance (CL_2-com_), steady-state volume of distribution (V_ss_), and the distribution rate constant from the peripheral to the central compartment (K_21_). Pseudo-random numbers were generated using NumPy version 1.26.4 (Python Software Foundation, https://www.python.org) in Python version 3.11.7. CL_cr_ was sampled from a truncated normal distribution, while CL_2-com_, V_ss_, and K_21_ were sampled from log-normal distributions. The distribution rate constant from the central to the peripheral compartment (K_12_) was fixed. From the 5000 generated parameter sets, 3652 subjects with CL_cr_ values between 30 and 110 mL/min were selected for simulation. A two-compartment pharmacokinetic model was employed to simulate C_VCM_ under various dosing regimens stratified by CL_cr_, determined with reference to the renal function-based dosage recommendations in the Japanese TDM Guidelines [2]. FDs were set as follows: 1.5 g for 30 ≤ CL_cr_ < 50 mL/min, and 1.75 g for 50 ≤ CL_cr_ < 110 mL/min. The maintenance dose (MD) and dosing interval (τ) were as follows: 1.25 g every 12 h (q12 h) for 100 ≤ CL_cr_ < 110 mL/min, 1.0 g q12 h for 80 ≤ CL_cr_ < 100 mL/min, 0.75 g q12 h for 60 ≤ CL_cr_ < 80 mL/min, 0.5 g q12 h for 40 ≤ CL_cr_ < 60 mL/min, and 0.75 g every 24 h (q24 h) for 30 ≤ CL_cr_ < 40 mL/min. The infusion time (t_in_) was defined as 1 h for single doses ≤ 1.0 g, 1.5 h for single doses > 1.0 g and ≤ 1.5 g, and 2 h for single doses > 1.5 g. AUC from 96 to 120 h (AUC_2-com_) was calculated using the trapezoidal rule based on the obtained C_VCM_-time profile. Subsequently, the C_peak,first_ and C_trough,ss_ at 120 h were extracted from the C_VCM_-time profile (Fig. S1). The Ke and Vd were determined using the solver function in Microsoft Excel (Microsoft Corporation, Redmond, WA, USA) based on Eqs. (1) and (2) of the one-compartment model [13, 14]. The AUC (AUC_1-com_) was calculated using Eq. (3) [9], with the Ke and Vd values.

1 $${C}_{peak,first}=\frac{FD}{{K}_{e}\cdot Vd\cdot {\mathrm{t}}_{in} }\cdot \left(1-{e}^{-{K}_{e}\cdot {t}_{in} }\right)\cdot {e}^{-{K}_{e}\cdot 1}$$

2 $${C}_{trough,ss}=\frac{MD}{{K}_{e}\cdot Vd\cdot {\mathrm{t}}_{in}}\cdot \frac{1-{e}^{-{K}_{e}\cdot {\mathrm{t}}_{in}}}{1-{e}^{-{K}_{e}\cdot\uptau }}\cdot {e}^{-{K}_{e}\cdot \left(\uptau -{\mathrm{t}}_{in}\right)}$$

3 $${AUC}_{1-com}=\frac{MD}{{K}_{e}\cdot Vd}\cdot \frac{24}{\uptau }$$

Subsequently, Vd_,app_ was calculated using Eq. (4), and alternative Ke (Ke_,alt_) was calculated from the obtained Vd_,app_ and C_trough,ss_ values based on Eq. (5) using the solver function in Microsoft Excel. The alternative AUC (AUC_1-com,alt_) was calculated from Vd_,app_ and Ke_,alt_ using Eq. (6).

4 $${Vd}_{, app}=\frac{FD}{{C}_{peak,first}}$$

5 $${C}_{trough,ss}=\frac{MD}{{K}_{e,alt}\cdot {Vd}_{,app}\cdot {\mathrm{t}}_{in}}\cdot \frac{1-{e}^{-{K}_{e,alt}\cdot {\mathrm{t}}_{in}}}{1-{e}^{-{K}_{e,alt}\cdot\uptau }}\cdot {e}^{-{K}_{e,alt}\cdot \left(\uptau -{\mathrm{t}}_{in}\right)}$$

6 $${AUC}_{1-com,alt}=\frac{MD}{{K}_{e,alt}\cdot {Vd}_{,app}}\cdot \frac{24}{\uptau }$$

Regression lines and correlations between AUC_1-com_ and AUC_2-com_ and between AUC_1-com,alt_ and AUC_2-com_ were assessed, and the relationship between the ratio of Vd_,app_ to Vd (Vd_,app_/Vd) and CL_2-com_ was visually examined.

### Creation of AUC nomograms

AUC nomograms were constructed for each dosing regimen, with Vd on the horizontal axis and C_trough,ss_ on the vertical axis, with the intersection representing the AUC. The dosing regimens comprised 15 different combinations of doses (0.5, 0.75, 1.0, 1.25, and 1.5 g) and τ values (8, 12, and 24 h). Vd ranged from 20 to 80 L in 5 L increments, and C_trough,ss_ ranged from 5 to 25 μg/mL in 1.0 μg/mL increments. Ke was calculated using Eq. (2) based on the dosing regimens, Vd, and C_trough,ss_. AUCs were derived using Eq. (3). Ke was estimated using the solver function of Microsoft Excel.

### Evaluation of AUC estimated using Vd_,app _and AUC nomograms

**Fig. 1:**
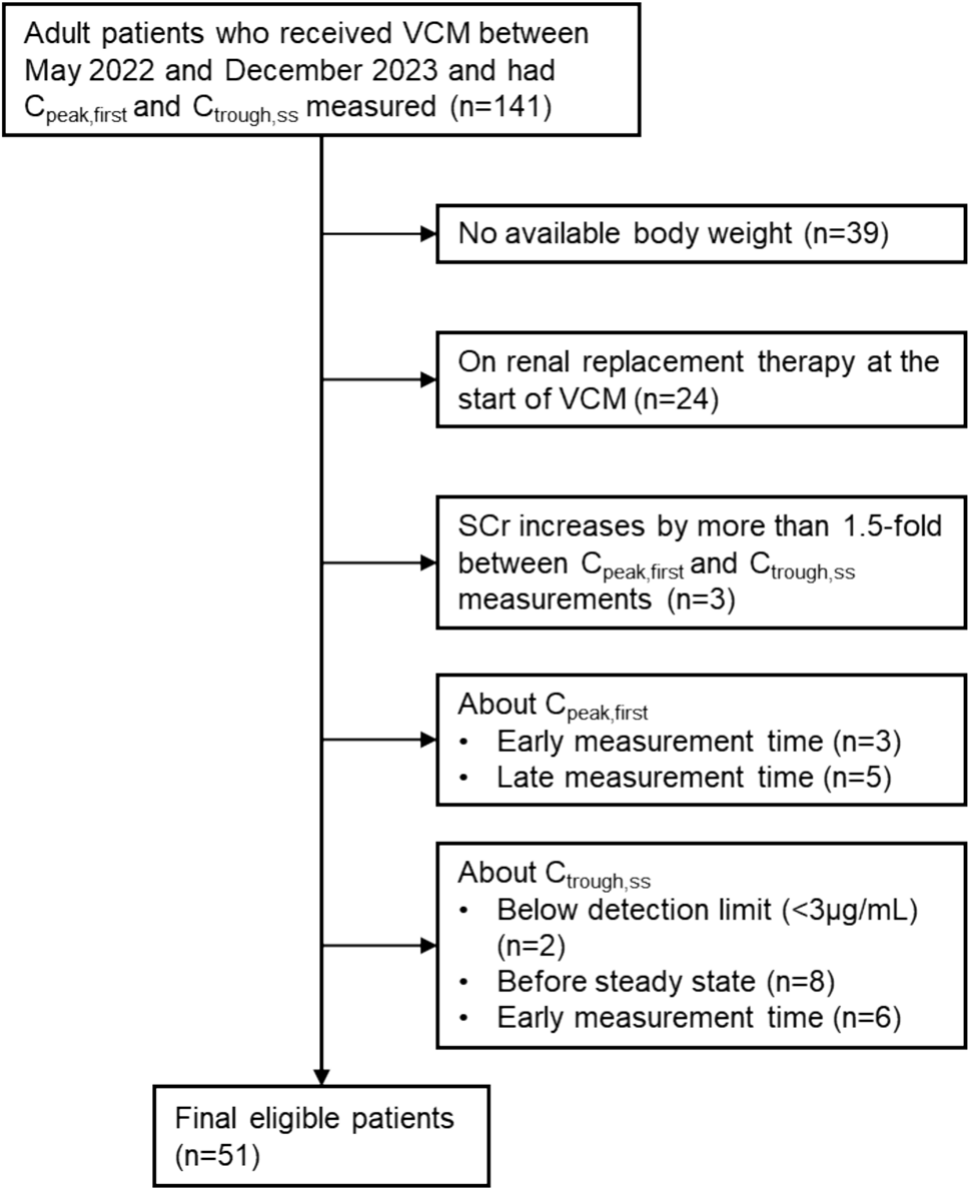
Flow chart of patient selection. Abbreviations: VCM, vancomycin; C_peak,first_, serum vancomycin concentration measured 1 h after completion of the first dose infusion; C_trough,ss_, trough concentration of vancomycin at steady state; SCr, serum creatinine

Adult patients who received VCM between May 2022 and December 2023, with C_peak,first_ and with C_trough,ss_ measured after a minimum of three doses, including the FD were enrolled. The exclusion criteria were: unknown BW; undergoing renal replacement therapy at VCM initiation; C_peak,first_ measured < 0.5 h or > 2 h after FD completion; C_trough,ss_ below the detection limit; C_trough,ss_ measured more than 1 h before the next dose; or serum creatinine (SCr) exceeding 1.5 times the SCr at C_peak,first_ measurement (Fig. 1). Age, sex, BW, SCr, CL_cr_, FD, MD, C_peak,first_, C_trough,ss_, and infectious disease type were recorded. CL_cr_ was estimated using the Cockcroft–Gault equation [15], as in Eq. (7).

7 $${CL}_{\mathrm{cr}}=\frac{\left(140-{\mathrm{age}}\right)\times \mathrm{body\;weight (kg)}}{72\times \mathrm{SCr}\mathrm{ (mg}\mathrm{/}\mathrm{dL)}}\times \left(0.85\hspace{0.17em}\text{if female}\right)$$

Subsequently, the AUC was calculated utilising three methods. First, employing C_peak,first_ and C_trough,ss_, CL (CL_Bayes_) and AUC (AUC_Bayes_) were estimated based on a two-compartment model using the Bayesian approach, with the web application PAT [16]. We selected the Yasuhara model as the PopPK parameters [12]. Second, based on the intravenous infusion equations of the one-compartment model using C_peak,first_ and C_trough,ss_, Ke, Vd, and AUC (AUC_LS_) were calculated using the least-squares method employing BMs-Pod version 8.06 (https://bmspod.web.fc2.com/), a PK analysis software based on Microsoft Excel. Third, Vd_,app_ was calculated from the FD and C_peak,first_ according to Eq. (4). Utilising the Vd_,app_ and C_trough,ss_, the closest values of Vd and C_trough,ss_ on the AUC nomograms were identified, and the corresponding intersection point was applied to determine the AUC (AUC_nmg_). Regression lines and correlations of AUC_LS_ and AUC_nmg_ relative to AUC_Bayes_ were evaluated. Furthermore, the concordance of AUC_nmg_ and AUC_LS_ with AUC_Bayes_ was assessed for the AUC classified into three categories as follows: AUC < 400, 400 ≤ AUC ≤ 600, and AUC > 600.

### Method of C_VCM_ measurement

The C_vcm_ was measured using a chemiluminescent immunoassay on the ARCHITECT^®^ analyzer i2000 SR (Abbott Japan Co., Ltd., Tokyo, Japan), with a lower limit of detection of 3 μg/mL.

### Statistical analysis

Statistical analyses were conducted using R version 4.1.1 (R Foundation for Statistical Computing, Vienna, Austria; http://www.R-project.org). Regression analyses between AUCs and between Vd and Vd_,app_ were performed using mcr package version 1.3.3.1, with regression lines determined by the Passing–Bablok method, while correlations were assessed using Kendall’s rank correlation coefficient (τb). The concordance of classifications was evaluated using the psych package version 2.4.12 with weighted kappa coefficients.

## Results

### Simulation with pseudo-parameters

The regression lines of AUC_1-com_ and AUC_1-com,alt_ against AUC_2-com_ were represented as y = 1.016x −10.47 (τb = 0.94) and y = 1.013x−27.19 (τb = 0.91), respectively. Both AUC_1-com_ and AUC_1-com,alt_ exhibited an approximately linear relationship with AUC_2-com_, with slopes approaching unity, demonstrating a strong correlation (Fig S2). Conversely, while Vd and Vd_,app_ also exhibited a strong correlation, the ratio of Vd_,app_/Vd increased with increasing CL_2-com_ (Fig. S3).

### AUC nomograms

**Fig. 2:**
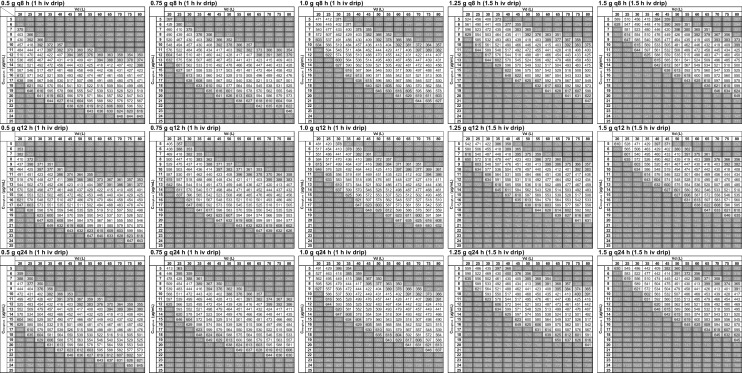
Nomograms for the area under the concentration–time curve (AUC nomograms). These nomograms are derived from the intravenous infusion equation of a one-compartment model. The volume of distribution (Vd) is plotted on the horizontal axis, and the trough concentration at steady state (C_trough,ss_) is plotted on the vertical axis. Their intersection indicates the corresponding AUC value. The white area represents 400 ≤ AUC ≤ 600, the light gray area represents 350 ≤ AUC < 400 or 600 < AUC ≤ 650, and the dark gray area represents AUC < 350 or AUC > 650

AUC nomograms (Fig. 2) showed that AUCs remained almost identical across different dosing intervals, provided that Vd, and C_trough,ss_ were consistent.

### Evaluation of AUC_nmg _estimated using Vd_,app _and AUC nomograms

**Fig. 3:**
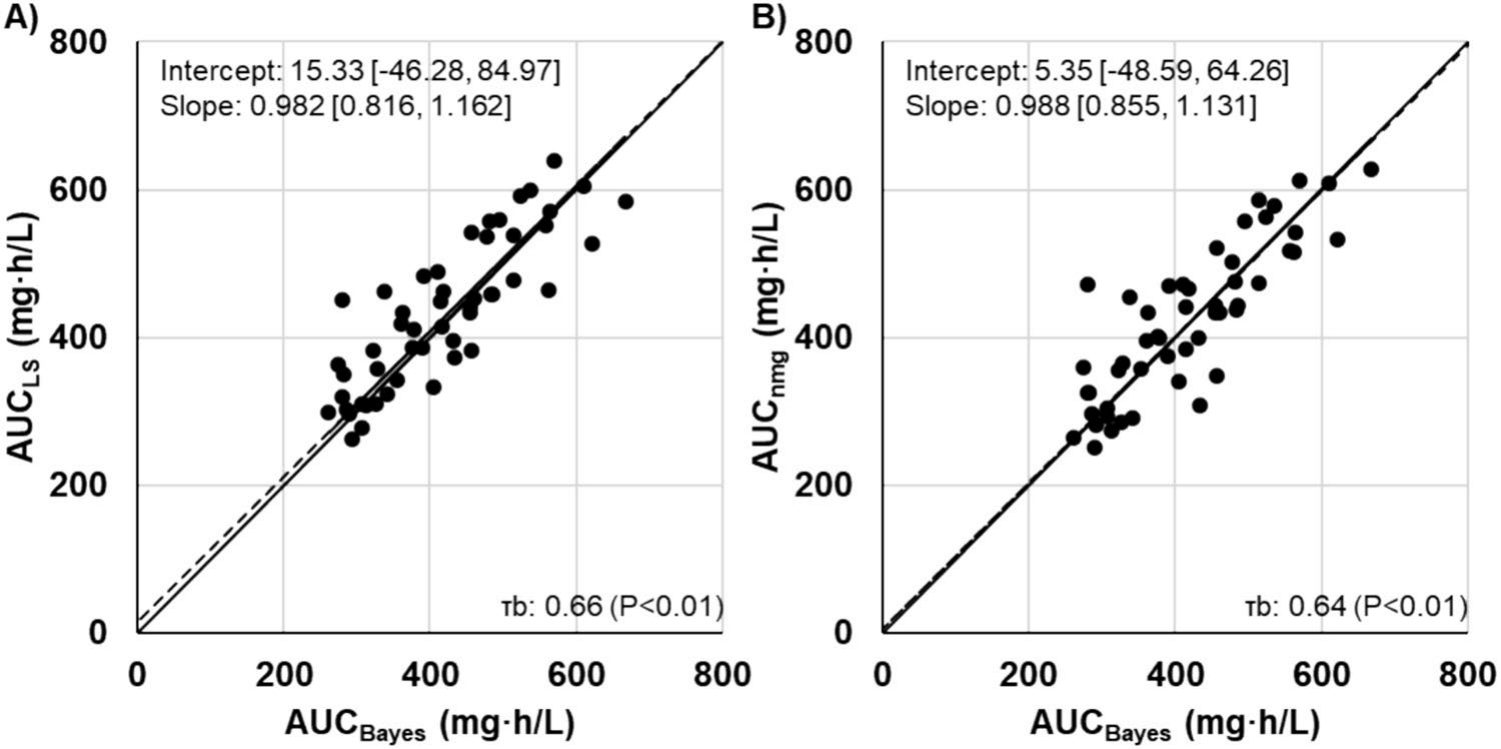
Correlations between area under the concentration–time curve (AUC) estimates (*n* = 51). (A) Correlation between the AUC estimated using a two-compartment model with the Bayesian approach (AUC_Bayes_) and the AUC estimated using a one-compartment model with the least-squares method (AUC_LS_). (B) Correlation between AUC_Bayes_ and the AUC estimated using AUC nomograms (AUC_nmg_). The solid line represents the identity line (Y = X), while the dotted line indicates the regression line obtained using the Passing–Bablok method. The slope and intercept are presented as values [95% confidence interval]. τb, Kendall’s rank correlation coefficient

**Fig. 4:**
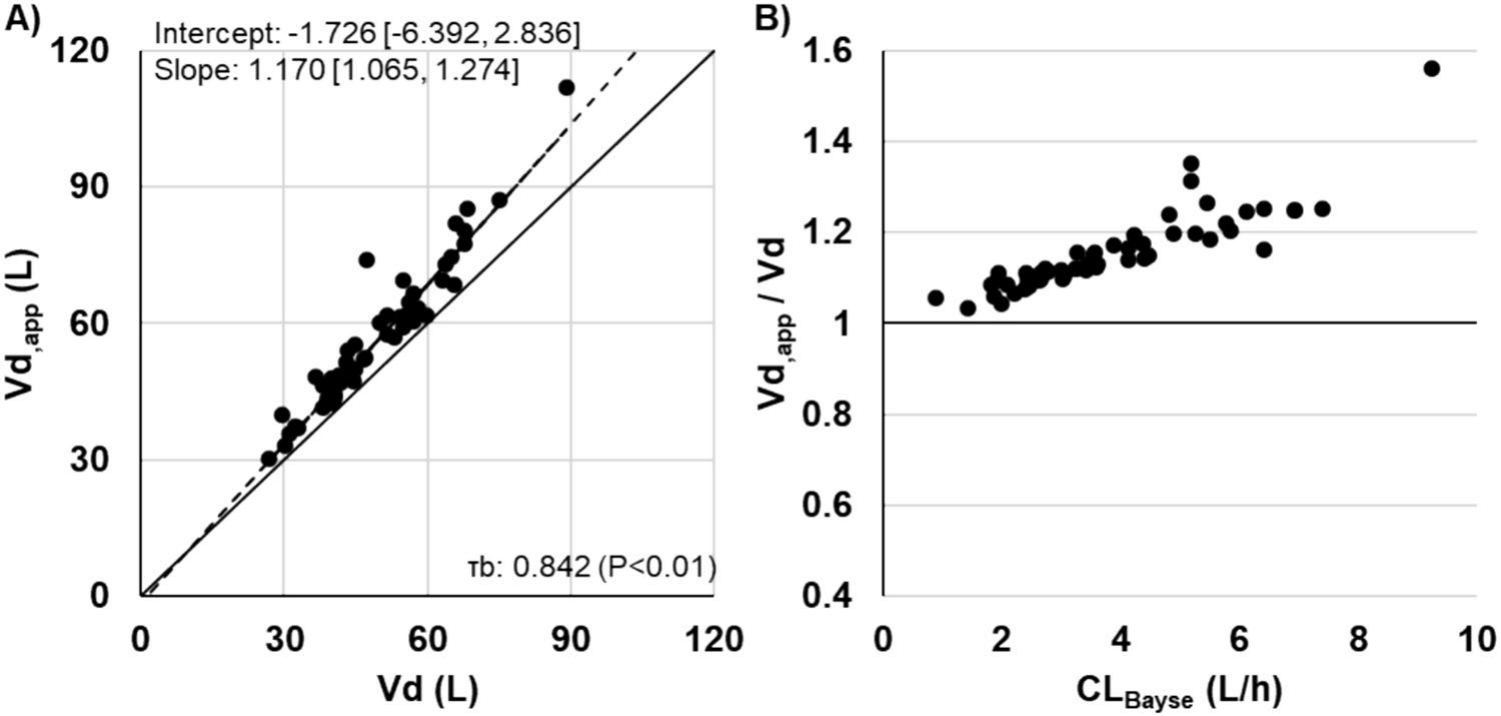
Relationship between the volume of distribution (Vd) estimated using a one-compartment model with the least-squares method and the apparent volume of distribution (Vd_,app_), as well as between Vd_,app_/Vd and the clearance estimated using a two-compartment Bayesian approach (CL_Bayes_) (*n* = 51). (A) Correlation between Vd and Vd_,app_. The solid line represents the identity line (Y = X), while the dotted line denotes the regression line obtained using the Passing–Bablok method. The slope and intercept are presented as values [95% confidence interval]. (B) Visual representation of the relationship between Vd_,app_/Vd and CL_Bayes_. τb, Kendall’s rank correlation coefficient

Patient characteristics are summarised in Table 1. All patients received a loading dose as the FD. Linear regression analysis revealed a strong positive correlation between AUC_Bayes_ with AUC_LS_ (y = 0.982x + 15.33, τb = 0.66) and AUC_nmg_ (y = 0.988x + 5.35, τb = 0.64), with slopes approximating 1 (Fig. 3). Moreover, the weighted kappa coefficients for concordance between AUC_Bayes_ and AUC_LS_ (0.56) and AUC_nmg_ (0.61), upon categorisation of AUC values into therapeutic ranges, indicated moderate to substantial agreement (Table 2). While Vd and Vd_,app_ exhibited a strong correlation, consistent with simulation-derived pseudo-parameters, the Vd_,app_/Vd ratio increased incrementally with increasing CL_Bayes_ (Fig. 4).

**Table 1:** Patient characteristics (*n* = 51)

Characteristic	
Age (years)	71 [61–78]
Sex (male/female)	32/19 (62.7%/37.3%)
Body weight (kg)	55.1 [47.5–69.4]
Serum creatinine (mg/dL)	0.65 [0.51–0.84]
Creatinine clearance (mL/min)^a^	88.3 [57.0–107.0]
First dose (mg/kg)	24.1 [21.5–27.3]
Maintenance dose (mg/kg/day)	27.0 [17.9–34.5]
C_peak, first_ (μg/mL)	25.3 [20.1–31.4]
C_trough, ss_ (μg/mL)	9.7 [8.2–13.0]
Infection type	
Respirato﻿ry infection	15 (29.4%)
Urinary tract infection	4 (7.8%)
Catheter-related bloodstream infection	3 (5.9%)
Bacteraemia	3 (5.9%)
Skin and soft tissue infection	2 (3.9%)
Bone and joint infection	2 (3.9%)
Burn	1 (2.0%)
Biliary tract infection	1 (2.0%)
Intra-abdominal infections	1 (2.0%)
Suspected catheter-related bloodstream infection	8 (15.7%)
Suspected meningitis	4 (7.8%)
Suspected infective endocarditis	1 (2.0%)
Unknown	6 (11.8%)

Data are presented as the median [interquartile range] or *n* (%).

^a^Creatinine clearance was estimated using the Cockcroft–Gault equation as shown below. $Creatinine\;clearance\;(mL/min)=\frac{\left(140-age\right)\times body\;weight(kg)}{72\times serum\;creatinine\;(mg/dL)}\times\left(0.85\;if\;female\right)$

Abbreviations: *C*_*peak,first*_, Serum vancomycin concentration measured 1 h after the completion of the first dose infusion;* C*_*trough,ss*_, Trough concentration of vancomycin at steady state

**Table 2:** Clinical classification agreement of AUC_LS_ and AUC_nmg_ with respect to AUC_Bayes_ (*n* = 51)

	**AUC**_**Bayes**__**(mg·h/L)**_				**Weighted kappa coefficient[95% CI]**
	**< 400**		**400–600**	**> 600**	
**AUC**_**LS**_** (mg·h/L)**					0.56 [0.36, 0.77]
** < 400**		17	4	0	
**400**–**600**		6	20	2	
** > 600**		0	1	1	
**AUC**_**ngm**_** (mg·h/L)**					0.61 [0.41, 0.81]
** < 400**		18	5	0	
**400**–**600**		5	19	1	
** > 600**		0	1	2	

*AUC*_*Bayes*_, area under the concentration–time curve using a two-compartment model with the Bayesian approach; *AUC*_*LS*_, area under the concentration–time curve using a one-compartment model with the least-squares method; *AUC*_*nmg*_, area under the concentration–time curve using AUC nomograms

## Discussion

In simulations utilising pseudo-parameters, AUC_1-com_ and AUC_1-com,alt_ achieved values comparable to AUC_2-com_, suggesting the feasibility of this method when PK parameters remain consistent from FD to C_trough,ss_ measurement. Additionally, AUC nomograms were developed based on the intravenous infusion equation of the one-compartment model (Eqs. 2 and 3) to estimate AUC from the dosing regimens, Vd, and C_trough,ss_, showing a strong correlation between AUC_nmg_ and AUC_Bayes_. The weighted kappa coefficient, which reflects the agreement rate of therapeutic ranges, was 0.61, indicating the practicality of the AUC nomograms for applicable patients. This approach is particularly beneficial in facilities with limited resources for Bayesian analysis or where AUC-guided TDM is not widely implemented [5, 6], and when obtaining C_vcm_ twice daily is challenging. Moreover, the accessibility and portability of AUC nomograms enable bedside evaluation, substantially reducing estimation time.

VCM PK parameters can be represented using either two- or one-compartment models. A mathematical approach based on PK parameters of the latter demonstrated comparable AUC values to a Bayesian approach [7]. One study comparing AUCs estimated by one- and two-compartment models with those from non-compartmental analysis in adult patients with stable renal function reported that the one-compartment model predicted AUC values 8.3% lower than the two-compartment model, although without clinically significant differences between the two models [17]. Furthermore, AUCs calculated using one- and two-compartment models based on C_trough,ss_ and C_vcm_ obtained at 1 h post-infusion (C_peak_) adequately reflected the true AUC, with both estimations being clinically reliable [18]. Overall, the one-compartment model provides comparable accuracy to the two-compartment model, with greater structural simplicity and ease of implementation. Our simulation results further confirmed its clinical feasibility.

Our AUC nomograms were constructed utilising Eq. (2) of the one-compartment model to determine C_trough,ss_ during intravenous infusion, deriving Ke from the combination of C_trough,ss_ and Vd. Although prior studies have proposed AUC calculation formulas [19] and nomograms [11] based on the intravenous administration equation, our AUC nomograms differ by t_in_. Both simplified calculation methods and nomograms offer high versatility and are advantageous for reducing input time compared to Bayesian approaches. Nevertheless, there are significant considerations regarding their utilisation. In our nomograms, C_trough,ss_ was presented at 1.0 μg/mL intervals and Vd at 5 L intervals, reflecting its intended application for approximate AUC estimation. Furthermore, the MD and dosing interval must remain constant until the C_trough,ss_ is measured, and the measured C_trough,ss_ must represent a steady-state value. Therefore, if these conditions are not met, the AUC nomograms should not be utilised; instead, a Bayesian approach accommodating variable doses and dosing intervals, should be employed; however, maintaining constant condition is generally considered safer by healthcare professionals; this is also a prerequisite for C_trough,ss_-guided TDM. Given our accumulated experience, we posit that this condition is not difficult to meet.

As demonstrated by the AUC nomograms, AUC_nmg_ values are comparable even with different dosing intervals, provided that the single dose, C_trough,ss_, and Vd remain constant. A larger single dose/Vd ratio is associated with a lower (< 10 μg/mL) C_trough,ss_ required to achieve therapeutic AUC. The single dose/Vd ratio can be converted to single dose/kg by substituting Vd normalised to BW, which corresponds to the previously reported nomogram of AUC based on single dose/kg and C_trough,ss_ [11]. This is an important consideration even at institutions employing the C_trough,ss_-guided TDM.

The utility of the C_trough,ss_-equation approach has been demonstrated, with high accuracy in AUC estimation [9, 10, 20]. The utilisation of C_trough,ss_ and the one-compartment model is consistent with the fundamental concept of this study; however, Vd determination remains a crucial factor. Previous studies have developed prediction equations for Vd, using the estimated Vd value along with C_trough,ss_ to calculate the AUC. Herein, we proposed an alternative approach in which Vd_,app_ was derived from C_peak,first_ and used as a surrogate for Vd. The C_peak,first_ following the FD possesses two key implications. First, it enables the evaluation of the patient’s PK parameters by comparing the derived Vd_,app_ with previously reported Vd prediction equations, such as Vd = 0.7 L/kg [19], Vd = 0.29 × age + 0.33 × BW + 11 [9], and Vd = 21.9 + 0.53 × BW [10]. Second, it serves as a means of verifying whether the increase in C_VCM_ following the FD falls within the expected range.

The AUC_nmg_ estimated using Vd_,app_ and the AUC nomograms in this study demonstrated a strong correlation with AUC_Bayes_, indicating the potential clinical applicability of this approach. However, several caveats regarding Vd_,app_ should be considered. The slope of the regression line [95% CI] for Vd versus Vd_,app_ was 1.166 [1.152, 1.180] in simulations using pseudo-parameters and 1.170 [1.065, 1.274] in actual clinical data, indicating comparable values. However, Vd_,app_ was approximately 17% greater than Vd. This discrepancy arises because Vd_,app_ is derived from the C_peak,first_ rather than the maximum C_VCM_ immediately following FD completion (C_first max_), suggesting that Vd_,app_ may inherently overestimate Vd. Additionally, Vd_,app_/Vd increased with CL_2-com_ and CL_Bayes_. This may be attributable to the recommended timing for measuring C_peak_, which is 1–2 h after administration, during the post-distribution phase. During this interval, higher clearance leads to more pronounced drug elimination, thereby amplifying the difference between C_first max_ and C_peak,first_. Therefore, the discrepancy between Vd and Vd_,app_ is expected to increase in patients with normal renal function and augmented renal clearance (ARC) [21]; caution should be exercised when using Vd_,app_ in such cases.

This study has some limitations. First, this study followed single-centre retrospective study with a limited sample size. Second, while AUC_Bayes_ was utilised as a comparator for AUC_nmg_ evaluation, it should ideally be compared with the true AUC obtained using the trapezoidal method based on multiple C_VCM_ values. Indeed, the AUC_Bayes_ can exhibit an error margin of ± 20% compared to the true AUC [2, 16], which necessitates further investigation. Third, the AUC_Bayes_ reflects estimates based on the parameters of the Yasuhara model [12], and has not been validated using other PopPK models. This is important as the accuracy may vary depending on the model employed [22]. Fourth, the patient population used for the validation of AUC_nmg_ in this study was biased towards elderly individuals with low BW and relatively preserved renal function (interquartile ranges: age, 61–78 years; BW, 47.5–69.5 kg; CL_cr_, 57–107 mL/min). Therefore, the accuracy of AUC_nmg_ may not be entirely maintained in specific populations, such as younger patients, obese patients, and those with severe renal impairment. Fifth, the measured C_peak,first_ and C_trough,ss_ values ranged only from 20.1 to 31.4 μg/mL and 8.2 to 13.0 μg/mL, respectively; validation across a broader concentration range is therefore warranted. Sixth, although we demonstrated that the Vd_,app_/Vd ratio increased proportionally with CL, we were unable to analyse the factors associated with elevated CL in individual patients. The highest Vd_,app_/Vd ratio observed in this study was 1.56, which was in a burn patient. Burn patients have been reported to exhibit higher clearance and ARC compared with non-burn patients [21, 23]. Other populations reported to exhibit ARC include patients with sepsis, febrile neutropenia, traumatic brain injury, and subarachnoid haemorrhage [21]. In these populations, Vd_,app_ may be unreliable depending on the degree of CL augmentation; as such, further research is warranted.

## Conclusion

Overall, we showed that the AUC_nmg_ estimated using the AUC nomograms and Vd_,app_ demonstrates closely alignment with the AUC_Bayes_, indicating its potential as a clinically feasible and straightforward methodology for evaluating AUC beyond the steady state based on C_peak,first_ and C_trough,ss_ measurements. While the inclusion of C_peak,first_ measurement and AUC nomogram verification represents an additional step compared with the conventional C_trough,ss_-based method, this approach is expected to be readily adoptable in clinical practice and contribute to the optimised utilisation of VCM. However, its applicability should be carefully evaluated, especially in patients with high clearance, such as those with ARC.

## Supplementary Information

Below is the link to the electronic supplementary material.

ESM 1 (DOCX 117 KB)

## Data Availability

Data is provided within the manuscript or supplementary information files.
