# Supplementary material for: Use of nomograms based on the one-compartment model in the estimation of area under the blood concentration–time curve of vancomycin: a retrospective cohort study
Source: Eur J Clin Pharmacol. 2025 Aug 4;81(11):1583–91. doi: 10.1007/s00228-025-03885-9 (PMC12511142; doi:10.1007/s00228-025-03885-9)
Supplement: Supplementary file 1 — (DOCX 117 KB) [file 228_2025_3885_MOESM1_ESM.docx]

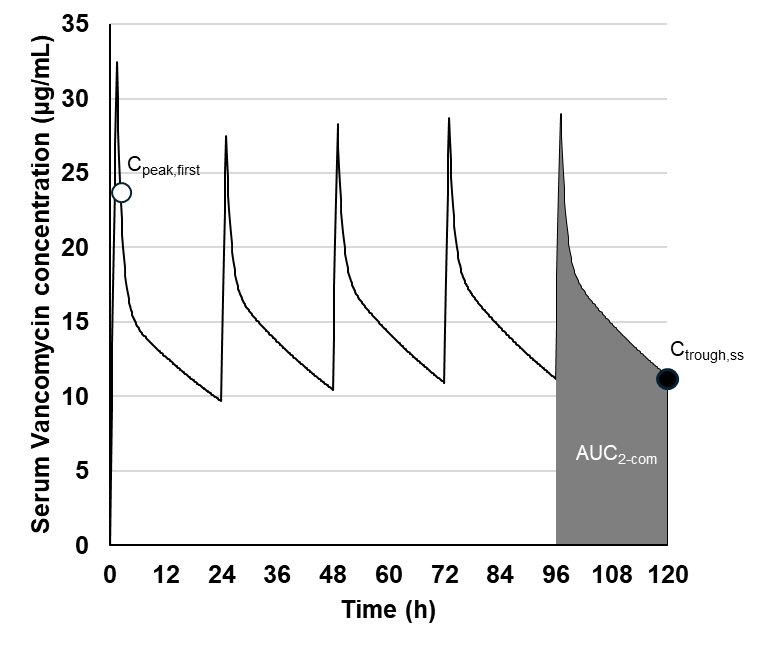


**Fig. S1** Simulation using pseudo-parameters based on the two-compartment model

The solid line represents the concentration–time profile, and the gray area represents the area under the concentration–time curve (AUC_2-com_). The white circle indicates the peak concentration obtained 1 h after the completion of the first dose infusion (C_peak,first_), while the black circle represents the trough concentration at steady state (C_trough,ss_).


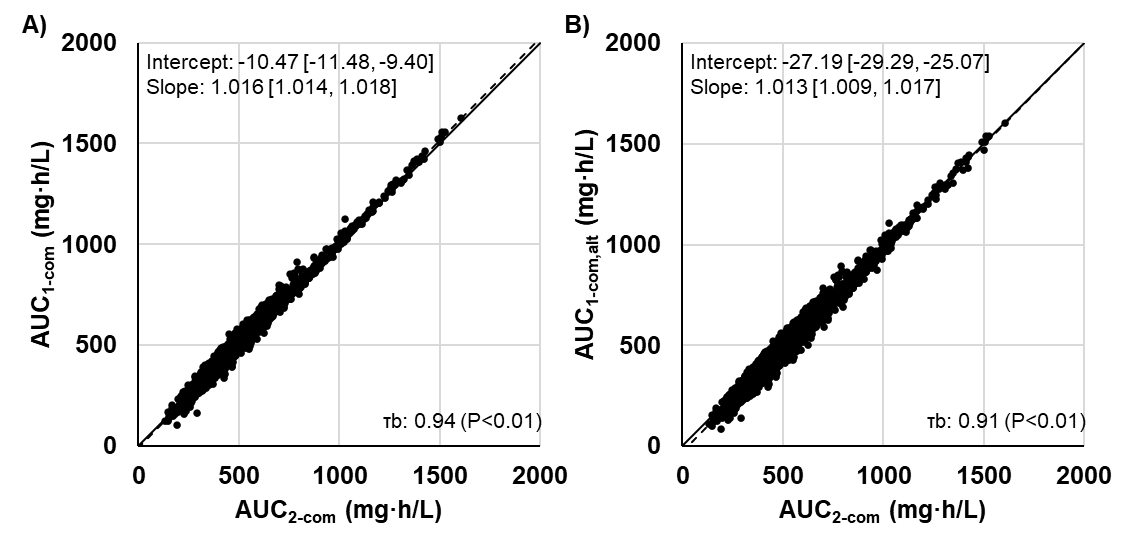


**Fig. S2** Correlations between area under the concentration–time curve (AUC) estimates in simulations using pseudo-parameters (n=3652)

A) Correlation between the AUC of the two-compartment model (AUC_2-com_) and the AUC of the one-compartment model (AUC_1-com_).

B) Correlation between AUC_2-com_ and the AUC of the one-compartment model calculated using the apparent volume of distribution (AUC_1-com,alt_).

The solid line represents the identity line (Y = X), while the dotted line indicates the regression line obtained using the Passing–Bablok method. The slope and intercept are presented as values [95% confidence interval].

τb, Kendall's rank correlation coefficient


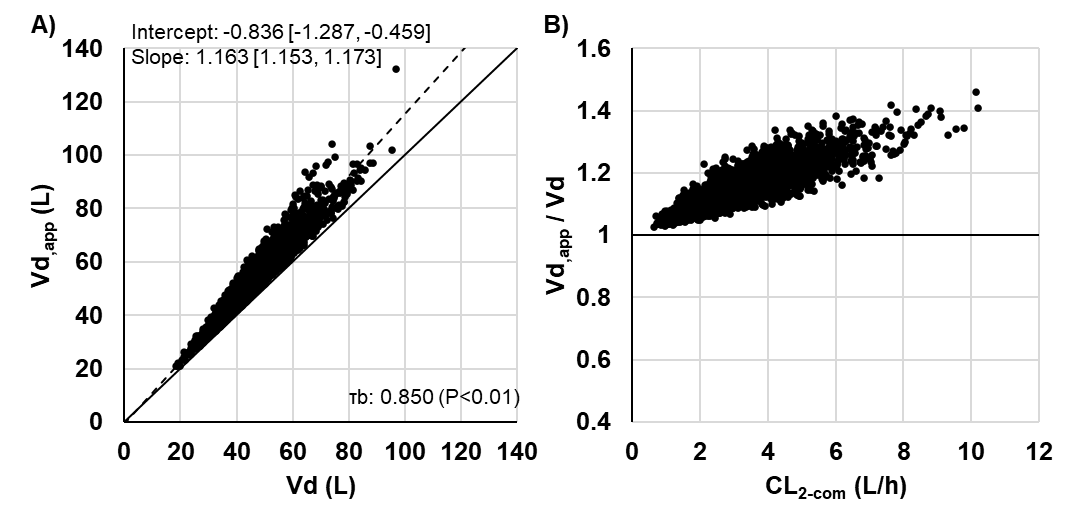


**Fig. S3** Relationship between the volume of distribution (Vd) and the apparent volume of distribution (Vd_,app_), as well as between Vd_,app_/Vd and the clearance in the two-compartment model (CL_2-com_), in the simulation using pseudo-parameters (n=3652)

A) Correlation between Vd and Vd_,app_. The solid line represents the identity line (Y = X), while the dotted line denotes the regression line obtained using the Passing–Bablok method. The slope and intercept are presented as values [95% confidence interval].

B) Visual representation of the relationship between Vd_,app_/Vd and CL_2-com_.

τb, Kendall's rank correlation coefficient
